# Aberrations in ion channels interacting with lipid metabolism and epithelial–mesenchymal transition in esophageal squamous cell carcinoma

**DOI:** 10.3389/fmolb.2023.1201459

**Published:** 2023-07-17

**Authors:** K. T. Shreya Parthasarathi, Susmita Mandal, John Philip George, Kiran Bharat Gaikwad, Sruthi Sasidharan, Seetaramanjaneyulu Gundimeda, Mohit Kumar Jolly, Akhilesh Pandey, Jyoti Sharma

**Affiliations:** ^1^ Institute of Bioinformatics, International Technology Park, Bangalore, India; ^2^ Manipal Academy of Higher Education (MAHE), Manipal, Karnataka, India; ^3^ Center for BioSystems Science and Engineering, Indian Institute of Science, Bangalore, India; ^4^ Department of Laboratory Medicine and Pathology, Rochester, MN, United States; ^5^ Center for Molecular Medicine, National Institute of Mental Health and Neurosciences (NIMHANS), Bangalore, India; ^6^ Center for Individualized Medicine, Rochester, MN, United States

**Keywords:** esophageal cancer, RNA-seq, membrane proteins, fatty acids, interaction networks

## Abstract

Esophageal squamous cell carcinoma (ESCC) is the most prevalent malignant gastrointestinal tumor. Ion channels contribute to tumor growth and progression through interactions with their neighboring molecules including lipids. The dysregulation of membrane ion channels and lipid metabolism may contribute to the epithelial–mesenchymal transition (EMT), leading to metastatic progression. Herein, transcriptome profiles of patients with ESCC were analyzed by performing differential gene expression and weighted gene co-expression network analysis to identify the altered ion channels, lipid metabolism- and EMT-related genes in ESCC. A total of 1,081 differentially expressed genes, including 113 ion channels, 487 lipid metabolism-related, and 537 EMT-related genes, were identified in patients with ESCC. Thereafter, EMT scores were correlated with altered co-expressed genes. The altered co-expressed genes indicated a correlation with EMT signatures. Interactions among 22 ion channels with 3 hub lipid metabolism- and 13 hub EMT-related proteins were determined using protein–protein interaction networks. A pathway map was generated to depict deregulated signaling pathways including insulin resistance and the estrogen receptor-Ca^2+^ signaling pathway in ESCC. The relationship between potential ion channels and 5-year survival rates in ESCC was determined using Kaplan–Meier plots and Cox proportional hazard regression analysis. Inositol 1,4,5-trisphosphate receptor type 3 (*ITPR3*) was found to be associated with poor prognosis of patients with ESCC. Additionally, drugs interacting with potential ion channels, including *GJA1* and *ITPR3*, were identified. Understanding alterations in ion channels with lipid metabolism and EMT in ESCC pathophysiology would most likely provide potential targets for the better treatment of patients with ESCC.

## 1 Introduction

Esophageal squamous cell carcinoma (ESCC) is the most prominent subtype of esophageal cancer worldwide, with a significant increasing incidence rate (Abnet et al., 2018). Several prognostic strategies have been introduced to improve the survival rates of patients with ESCC (Hirano and Kato, 2019). Hitherto, negative results from clinical trials of targeted therapies suggest the need to identify novel biomarkers in regulating significant molecular mechanisms of ESCC progression (Hirano and Kato, 2019). Alterations in ion channels, the membrane proteins that allow the influx and efflux of ions across membranes, have been reported in several tumors (Sharma et al., 2019; Parthasarathi et al., 2022; Aydar et al., 2009; Chioni et al., 2010). Roles of ion channels in apoptosis and their potential in cell regulation and tumor progression were identified (Comes et al., 2015; Fraser et al., 2014; Bustin et al., 2001). The voltage-gated K^+^, Cl^−^, and Ca^2+^, and transient receptor potential (TRP) channels contributed in the progression of several tumors (Shiozaki et al., 2021; Litan and Langhans, 2015). Potassium voltage-gated channels, KCNA1 (Northcott et al., 2012) and KCNA3 (Comes et al., 2013), have been reported to be overexpressed in different malignancies such as medulloblastoma, breast, colon, and prostate cancers. In addition, the growth and migration of glioma cells were observed as regulated by the upregulation of splice variants of large potassium channels (Liu et al., 2002).

Recently, it has been reported that EMT leads to changed plasma membrane ion channel expression (Azimi and Monteith, 2016). Aquaporins, Cl^−^ and Ca^2+^ channels, were found to be involved in the progression of ESCC (Shiozaki et al., 2021). Furthermore, the progression of esophageal cancer was identified to be regulated by different types of ion channels, including voltage-gated K^+^, Cl^−^, and Ca^2+^, and TRP channels expressed by esophageal cancer cells and tissues (Shiozaki et al., 2021).

Alterations in lipid metabolism would result in variations in membrane composition, protein distribution, and cellular functions. This further results in the development and progression of various diseases, including cystic fibrosis, neurological disorders, and cancer (Huang and Freter, 2015). Furthermore, various studies indicate the role of lipids in enhancing migration and proliferation of tumor cells (Wang et al., 2021a; Deng et al., 2019; Kouba et al., 2019). The lipid metabolism-related genes, such as *CYP2C9*, *DGAT1*, *UGT1A6*, *INS*, *HPGDS*, and *LPL*, were identified as prognostically significant in patients with lung adenocarcinoma (Li et al., 2020). Chen and colleagues reported preoperative serum total cholesterol and low-density lipoprotein cholesterol as prognostic factors in ESCC patients who underwent esophagectomy (Chen et al., 2017). Moreover, cellular lipid metabolism involves ion channels and may influence the functions of other ion channels. The conversion of phosphatidylinositol bisphosphate to diacylglycerol or sphingomyelin to ceramide has been reported in both the maintenance of membrane surface potential and opening and closing of channel proteins (Kasimova et al., 2014), (Minke and Cook, 2002), (Huang and Freter, 2015).

The presence of ion channels, lipid metabolism- and EMT-related genes on the membrane enhances the possibility of various interactions between them and their role as a union in several key cellular processes. The alterations in the membrane potential may lead to changes in the lateral spatiotemporal distribution of charged lipids present in the membrane (Downward, 2003; Accardi, 2015). This could further affect the membrane-bound signaling proteins like Ras family GTPases (Downward, 2003; Accardi, 2015). Kouba et al. discussed on devising novel therapeutic strategies by understanding the inter-dependencies between Ca^2+^ and Ca^2+^-activated K^+^ channels with lipid mediators in epithelial ovarian cancer (Kouba et al., 2019). However, currently there is no study to indicate the relationship of ion channels with lipid metabolism-related genes and the EMT program in ESCC.

In this study, transcriptome sequencing (RNA-seq) data were obtained from 12 matched paired samples of patients with ESCC from the Kidwai Memorial Institute of Oncology, Bangalore, India (IOB-KMIO), and the publicly available dataset GSE32424 from the Gene Expression Omnibus (GEO) database was used to identify alterations co-expressions between ion channels, lipid metabolism- and EMT-related genes in ESCC. Thereafter, the deregulated genes were correlated with EMT using EMT scoring techniques. Subsequently, protein–protein interaction (PPI) network and clustering analyses were performed. Furthermore, the expression profiles of ESCC patients from The Cancer Genome Atlas (TCGA) and healthy individuals from Genotype-Tissue Expression (GTEx) data resources were used to compare the identified deregulated ion channels interacting with lipid metabolism- and EMT-related genes in a larger cohort of patients. In total, 1,081 unique genes were found to be differentially expressed in the transcriptome of patients with ESCC that included 113 ion channels, and 487 lipid metabolism- and 537 EMT-related genes. The interactome consisting of the interactions between the three sets of proteins that included 16 significantly altered ion channels was generated. Gap junction alpha-1 (GJA1), transient receptor potential cation channel subfamily V member 3 (TRPV3), and ITPR3 were found interacting with hub proteins AKT1, CCL2, CDH2, FN1, EGFR, and CAV1 and were a part of dense clusters within the PPI network. A pathway map was generated to depict the involvement of ion channels interacting with lipid metabolism- and EMT-related genes in various pathways in ESCC. Deregulation of several pathways, including insulin signaling and estrogen receptor-calcium signaling, was identified in ESCC. *ITPR3* was found to be prognostically significant. Additionally, drugs interacting with the potential ion channels were identified. The analyses could identify the importance of interactions between ion channels and lipid metabolism-related genes that might orchestrate the processes involved in EMT. Experimental validation of the potential ion channels is needed to further elucidate their roles as potential targets in ESCC.

## 2 Materials and methodology

### 2.1 Institutional Review Board statement

The study was approved by the Institutional Review Board Medical Ethics Committee of the Kidwai Memorial Institute of Oncology, Bangalore, India (KMIO/MEC/021/24 dated 30 November 2016), and conducted according to the guidelines of the Declaration of Helsinki.

### 2.2 Informed consent statement

Informed consent was obtained from all subjects involved in the study.

### 2.3 Data collection

#### 2.3.1 Gene list collection

The list of ion channels was downloaded from the HGNC database (accessed on 11th March 2022), lipid metabolism-related genes were collected from the literature (Li et al., 2020; Fernandez et al., 2020; Dunn et al., 2019; Moskot et al., 2015; Meng et al., 2021; El-Fasakhany et al., 2001; Russo et al., 2018; Lutz and Cornett, 2013; Astudillo et al., 2012; Sonnweber et al., 2018; Hanna and Hafez, 2018), and EMT-related genes, from dbEMT (v2.0). The total number of genes present in each gene category is provided in Supplementary Table S1.

#### 2.3.2 RNA-seq data collection

Twelve matched paired samples of patients with ESCC were collected from the Kidwai Memorial Institute of Oncology (IOB-KMIO), Bangalore, India. Supplementary File S1 contains the experimental procedure used for transcriptome sequencing. Another RNA-seq dataset, GSE32424, obtained using the Illumina Genome Analyzer IIx platform was downloaded from the GEO database. Furthermore, the gene expression profiles of samples corresponding to ESCC from the TCGA_ESCA cohort were downloaded from the UCSC Xena portal (Goldman et al., 2020) using R software (v4.1.3) UCSCXenaShiny library (Wang et al., 2021b). The healthy samples of the esophagus were downloaded from a GTEx data resource using the UCSCXenaShiny library. The detailed description on all the samples included in these datasets and the criteria used for the selection of healthy samples from GTEx is described in Supplementary Tables S2–S5. The RNA-seq datasets from IOB-KMIO and GSE32424 were used as the discovery datasets, and the TCGA-GTEx dataset was utilized to compare the deregulated genes in a larger cohort of patients with ESCC.

### 2.4 Data processing

The raw reads from IOB-KMIO and GSE32424 datasets were analyzed using the workflow depicted in Supplementary Figure S1. The raw reads were quality checked using FastQC, quality trimmed using Trimmomatic (v0.39), and clean reads were obtained. Furthermore, clean reads were mapped and aligned to the human genome GRCh38.105 using the STAR RNA-seq aligner (v2.7.10a) (Supplementary Tables S6, S7). Furthermore, the aligned reads were quantified using HTSeq (v1.99.2). R (v4.1) scripts were used to merge the HTSeq output files, and the R Bioconductor package, biomaRt (Durinck et al., 2009), was used to convert the Ensembl IDs to HGNC gene symbols. The ESCC RNA-seq log (read count +1) normalized data from TCGA were transformed to raw read counts using R scripts. Furthermore, the read counts corresponding to ion channels, lipid metabolism- and EMT-related genes were parsed using R scripts. Supplementary Figure S2 depicts the overall workflow used for the identification of ion channels and their interactions with lipid metabolism and EMT.

### 2.5 Identification of differentially expressed genes

Differentially expressed ion channels, lipid metabolism- and EMT-related genes in the IOB-KMIO and GSE32424 datasets were identified using the R Bioconductor package DESeq2 (v4.1.2) (Love et al., 2014). An adjusted *p*-value (*padj*) ≤ 0.05 and log2-fold change (log2FC) > 0.6 were set as the criteria for the selection of upregulated genes, and the genes with *p*adj ≤ 0.05 and log2FC < −0.6 were selected as downregulated genes. A non-redundant list with all DEGs obtained from the two datasets was generated.

### 2.6 Construction of co-expression networks of DEGs

The expression profiles of ESCC patients from IOB-KMIO and GSE32424 datasets corresponding to DEGs were parsed. Furthermore, these profiles were provided as input to the R Bioconductor package weighted gene co-expression network analysis (WGCNA) (Zhang and Horvath, 2005; Langfelder and Horvath, 2008) to construct the scale-free co-expression networks. The read counts provided as input were DESeq2-normalized. On the basis of a scale-free network model, an adjacency correlation matrix was calculated (Langfelder and Horvath, 2008). A suitable soft threshold power that ensured a scale-free network was selected based on the pickSoftThreshold function. Thereafter, a signed topological overlap matrix (TOM) and the corresponding dissimilarity matrix (1-TOM) were derived from the obtained adjacency matrix. The dissimilarity matrix was used for performing hierarchical clustering with the DynamicTreeCut algorithm (Langfelder et al., 2008) in order to group the genes with similar expression profiles into the same gene modules.

The expression profiles of each obtained modules were epitomized by module eigengenes (MEs) and correlated with binary trait (normal and tumor) values, and a relationship between the module and traits was obtained. The *p*-value was calculated as a confidence measure for the module–trait relationship. Furthermore, gene significance (GS) and module membership (MM) values were calculated to estimate the association of individual genes in the module with the binary trait and the correlation of gene expression profiles with MEs, respectively. The intramodular gene connectivity, with the binary trait, was estimated on the basis of GS vs. MM plots (Langfelder and Horvath, 2008). Thereafter, the most correlated modules with a significant *p*-value were selected from each dataset. The obtained weighted gene co-expression networks were exported to Cytoscape with the weight threshold value set to 0.02 for visualization.

### 2.7 Calculation of EMT scores

In this study, 76-gene EMT signature-based (GS76), multinomial logistic regression-based (MLR), and Kolmogorov–Smirnov test-based (KS) methods developed in the past (Chakraborty et al., 2020; George et al., 2017; Mandal et al., 2021) were used for the calculation of EMT scores of transcriptomic profiles of samples from patients with ESCC. A higher KS and MLR score indicated a more mesenchymal sample, and a higher GS76 score indicated a more epithelial sample. MLR quantifies the extent of EMT on a scale of [0, 2], and KS scores are defined on a scale of [−1, +1] (Chakraborty et al., 2020). The scatter plots between different EMT scores were plotted using the R package “ggplot2” (Wickham, 2016) with correlation coefficients and *p*-values calculated using the R package “corrplot” (Wei et al., 2017) by Pearson’s method.

### 2.8 Computation of the correlation of altered ion channels and lipid metabolism-related genes with EMT

The estimated EMT scores of IOB-KMIO samples by the GS76, MLR, and KS methods were correlated with the expression profiles of the module ion channels and lipid metabolism-related genes interacting with EMT-related genes in ESCC.

### 2.9 Construction of the protein–protein interaction networks of co-expressed gene modules

To infer the interactions between the genes in the modules, protein–protein interaction networks (PPINs) were constructed using the Search Tool for the Retrieval of Interacting Genes/Proteins (STRING) (v11.0) database (von Mering et al., 2003; Szklarczyk et al., 2021). The interactions with a medium confidence and false discovery rate of 1% were further visualized in Cytoscape (v3.9) (Shannon et al., 2003). The unconnected nodes in the network were discarded. Thereafter, NetworkAnalyser, a Cytoscape plugin, was used to calculate the various properties of the network, including degree centrality and betweenness centrality. The top 15 nodes in the network with the highest degree centrality were selected as the hub nodes in the networks.

### 2.10 Identification of highly interacting clusters within the protein–protein interaction networks

The Molecular Complex Detection (MCODE) (Bader and Hogue, 2003) tool, a Cytoscape plugin, was used to identify the dense clusters representing the highly interacting nodes within the PPI networks. The parameter maximum depth was set to 100 degree, k-core was maintained as 2, and the node score was set to 0.2. Thereafter, the clusters consisting of ion channels, and lipid metabolism- and EMT-related nodes were selected to identify ion channels interacting with both lipid metabolism- and EMT-related nodes.

### 2.11 Generation of the pathway map of ion channels interacting with lipid metabolism- and EMT-related genes

An extensive literature search was carried out using PubMed, and reactions like activations, inhibitions, and translocations that describe the interactions between the clustered ion channels, lipid metabolism- and EMT-related genes were annotated. Thereafter, a signaling pathway map consisting of the deregulated ion channels, lipid metabolism- and EMT-related genes was generated using PathVisio (version 3.3.0) in the Graphical Pathway Markup Language (GPML) format.

### 2.12 Association of putative ion channels in the survival of patients with ESCC

TCGA-ESCA dataset was used to compare the alterations in the ion channels obtained from IOB-KMIO and GSE32424 datasets. Differentially expressed ion channels, lipid metabolism- and EMT-related genes in the RNA-seq TCGA-GTEx ESCC dataset were identified using the R Bioconductor package DESeq2 (v4.1.2). An adjusted *p*-value (*padj*) ≤ 0.05 and log2FC > 0.6 were set as the criteria for the selection of upregulated genes, and the genes with *padj* ≤ 0.05 and log2FC < −0.6 were selected as downregulated genes.

Furthermore, the clinical data on patients with ESCC were extracted using the R Bioconductor package “RTCGA” for survival analysis. To determine the correlation between ion channel expression and a 5-year survival rate of patients with ESCC, Cox proportional hazard regression analysis was performed. The R Bioconductor package “survival” was utilized to calculate the hazard ratio (HR) with a confidence interval of 95% and the log rank *p*-values. Kaplan–Meier (KM) survival plots were generated to visualize the differences between the high and low expressions of the putative ion channels using the R Bioconductor package “survminer”. The median value was used as the group cut-off criteria.

### 2.13 Identification of drugs interacting with ion channels correlating with lipid metabolism- and EMT-related genes

Ion channels are potential clinical targets for restorative intervention due to their localization to the plasma membrane and different regulation mechanisms (Anderson et al., 2019). The list of known drugs interacting with all the human genes was collected from The Drug–Gene Interaction Database (DGIdb) (Freshour et al., 2021). This list was parsed with the list of potential ion channels interacting with lipid metabolism- and EMT-related genes.

## 3 Results

### 3.1 DEGs in IOB-KMIO and GSE32424 datasets

A total of 282 genes, including 39 ion channels, 108 lipid metabolism genes, and 135 EMT-related genes, were found as differentially expressed in the IOB-KMIO dataset. In total, 1,013 genes, including 89 ion channels, 433 lipid metabolism genes, and 491 EMT-related genes, were found differentially expressed in the GSE32424 dataset (Table 1 and Supplementary Table S8).

**TABLE 1:** Number of differentially expressed ion channels, and lipid metabolism- and EMT-related genes in IOB-KMIO and GSE32424 datasets.

	IOB-KMIO			GSE32424		
	Ion channels	EMT-related genes	Lipid metabolism-related genes	Ion channels	EMT-related genes	Lipid metabolism-related genes
Upregulated	7	50	30	54	281	235
Downregulated	32	85	78	35	210	198

**FIGURE 1:**
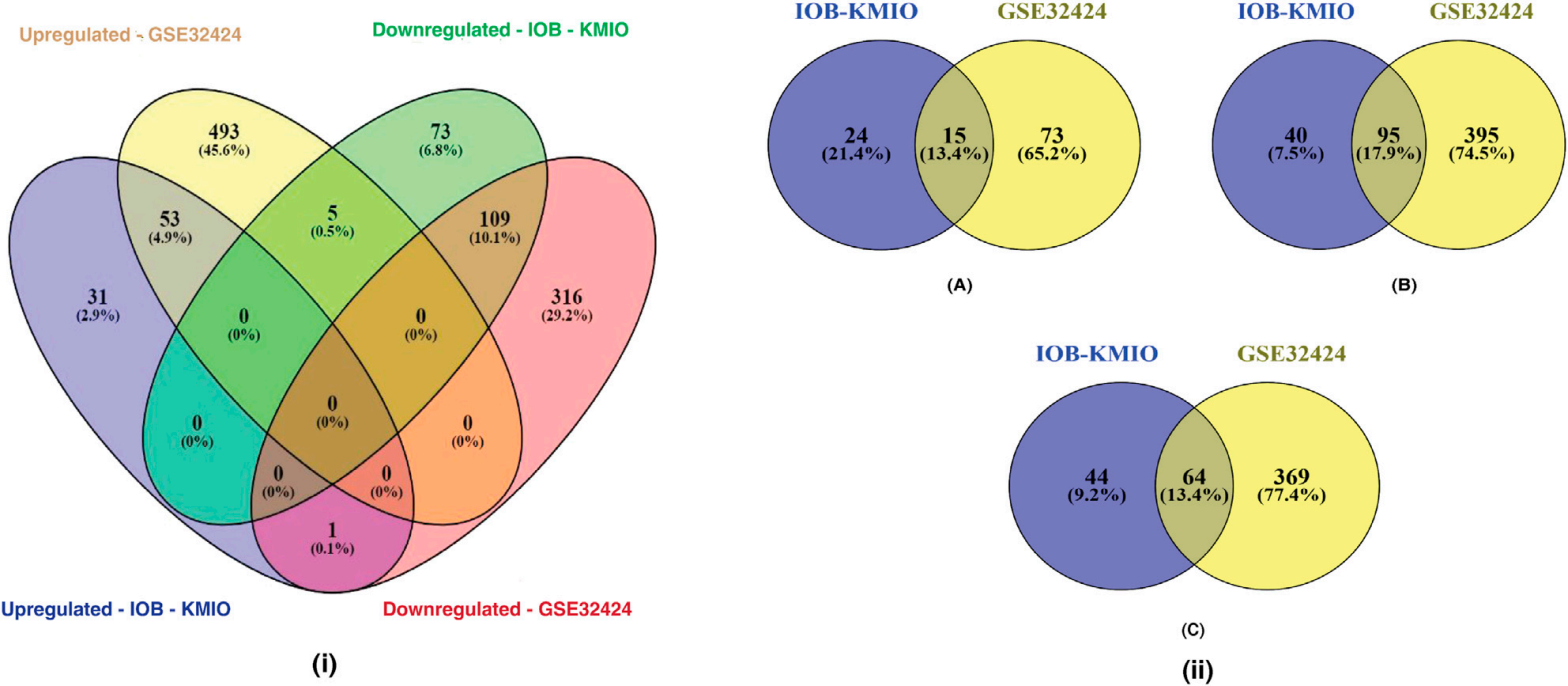
Venn diagrams depicting the overlaps of differentially expressed genes (DEGs). **(i)** Overlaps in the upregulated and downregulated genes (ion channels, lipid metabolism- and EMT-related genes) in IOB-KMIO and GSE32424 datasets; **(ii)** overlaps of DEGs among the datasets IOB-KMIO and GSE32424. **(A)** Overlaps in the deregulated ion channels, **(B)** overlaps in the deregulated EMT-related genes, and **(C)** overlaps in the deregulated lipid metabolism-related genes.

Figure 1(i) represents the overlaps between the upregulated and downregulated genes in IOB-KMIO and GSE32424 datasets. A total of 1,081 DEGs were identified. Figure 1(ii) represents the overlaps between ion channels, and lipid metabolism- and EMT-related genes across IOB-KMIO and GSE32424 datasets.

### 3.2 Co-expression networks of DEGs

**FIGURE 2:**
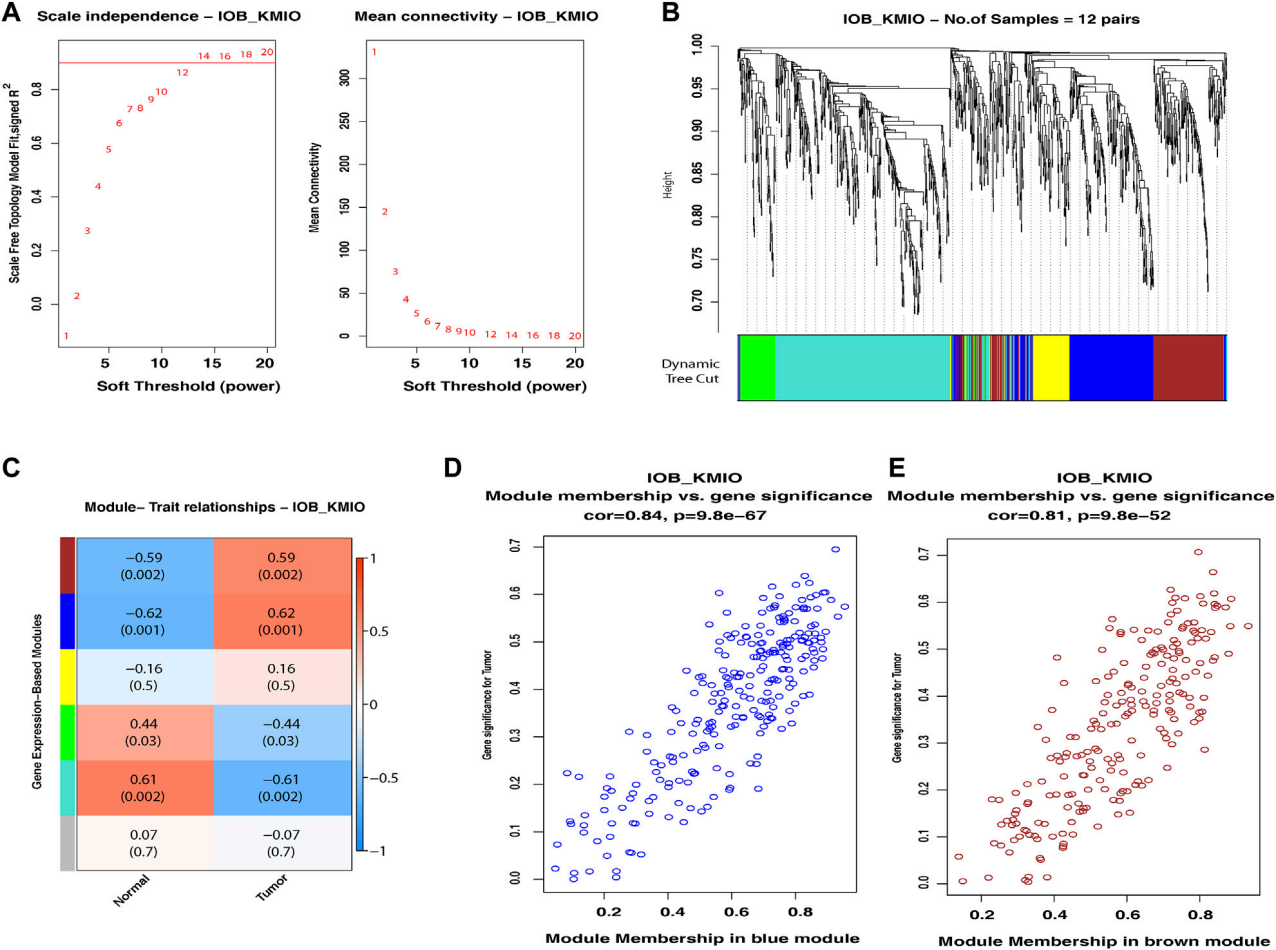
Weighted gene co-expression network analysis of the IOB-KMIO dataset. Gene modules with co-expressed ion channels, and lipid metabolism- and EMT-related gene modules based on non-redundant DEGs. **(A)** Soft-thresholding power: **(A)** a soft-thresholding power of 14 was chosen in the dataset IOB-KMIO to ensure a scale-free network model; **(B)** hierarchical clustering of genes into modules: the horizontal bar below the tree diagram represents the modules depicted by different colors. Five modules (turquoise, blue, yellow, brown, and green) were obtained; **(C)** correlation between module eigengenes and binary traits—normal and tumor. Rows correspond to modules, depicted as different colors, and columns are the binary traits. Numbers in each cell are the correlation coefficient between module eigengenes and the binary traits, and the corresponding *p*-value. **(D)** Blue, brown, and turquoise modules were chosen as significant modules. **(E)** Scatter plot of gene significance for the binary trait vs. the module membership in the selected modules. Blue and brown modules showed a better gene correlation within module gene correlation.

WGCNA of the identified DEGs led to the detection of gene modules consisting of genes co-expressed in each of the dataset. For the IOB-KMIO dataset, a soft thresholding power of 14 was chosen and the minimum module size of 50 with a deepSplit parameter set to 0 was used to construct co-expression networks. Similarly, for the GSE32424 dataset, a soft thresholding power of 18 was chosen and the minimum module size of 50 with a deepSplit parameter set to 0 was used to construct co-expression networks. The IOB-KMIO dataset was split into five modules. Based on the *p*-value and correlation values, the blue and brown modules were selected (Figure 2). GSE32424 was split into two modules—turquoise and blue. Both the modules showed a good correlation among the genes within the modules (Supplementary Figure S3).

### 3.3 Correlation of module genes with EMT

**FIGURE 3:**
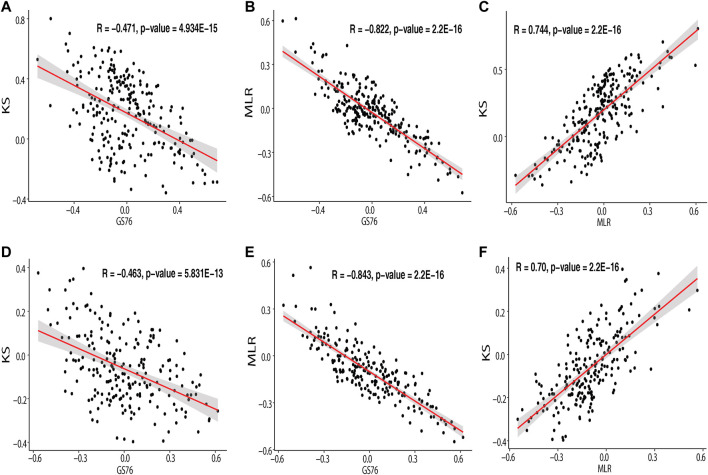
Scatter plot depicting the correlation of the module genes correlating with GS76, KS, and MLR EMT scores. **(A)** Correlation of IOB-KMIO blue module genes with GS76 and KS EMT scores; **(B)** correlation of IOB-KMIO blue module genes with GS76 and MLR EMT scores; **(C)** correlation of IOB-KMIO blue module genes with MLR and KS EMT scores; **(D)** correlation of IOB-KMIO brown module genes with GS76 and KS EMT scores; **(E)** correlation of IOB-KMIO brown module genes with GS76 and MLR EMT scores; **(F)** correlation of IOB-KMIO brown module genes with MLR and KS EMT scores.

The EMT scores obtained using GS76, MLR, and KS metrics for the samples in IOB-KMIO are provided in Supplementary Table S9. A negative GS76 score with a positive KS score and a higher MLR score indicates the sample to be a mesenchymal sample. Similarly, the sample with a positive GS76 score and a negative KS score with a lower MLR score could be an epithelial sample. The correlation between the scores corresponding to the respective method in the dataset was calculated and is depicted in Figure 3 and Supplementary Table 10. MLR and KS scores correlated positively with each other. GS76 scores correlated negatively with both MLR and KS scores.

### 3.4 Protein–protein interaction networks of co-expressed gene modules

**FIGURE 4:**
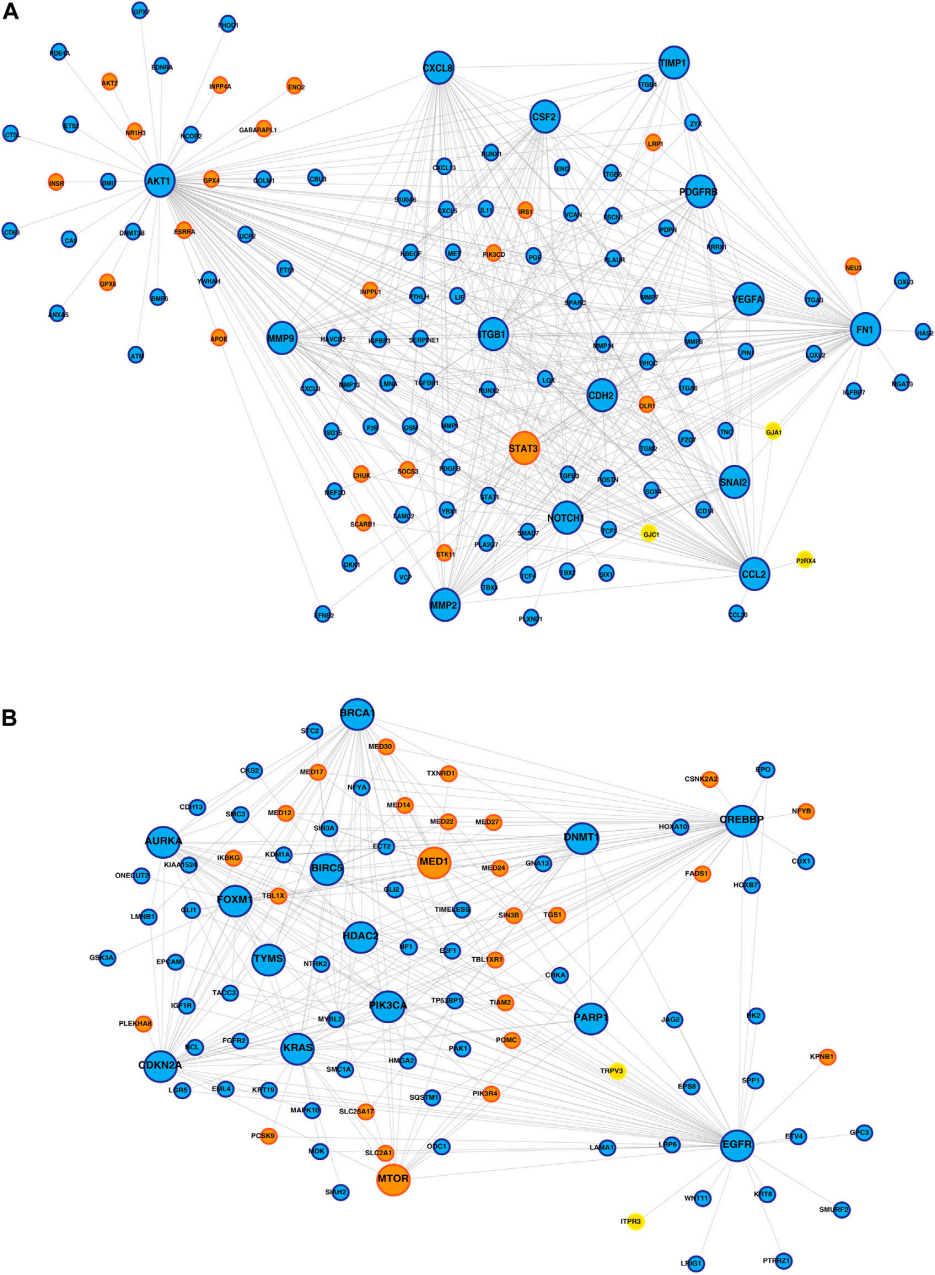
Protein–protein interaction network of ion channels—yellow nodes, lipid metabolism-related proteins—orange nodes, and EMT-related proteins—blue nodes. **(A)** PPIN depicting the proteins interacting with the hub nodes in the IOB-KMIO blue module network. **(B)** PPIN depicting the proteins interacting with the hub nodes in the IOB-KMIO brown module network.

PPI networks obtained through the STRING database for the co-expressed gene modules revealed the predicted PPIs between co-expressed DEGs (Supplementary Figure S4). The lipid metabolism- and EMT-related proteins with multiple interactions were identified as hub nodes in the network (Figure 4; Supplementary Figure S5). Several ion channels were found interacting with the hub nodes in the network. In the network generated from the blue module of the KMIO dataset, the ion channels GJA1, P2RX4, and GJC1 interacted with the hub nodes AKT1, CCL2, CDH2, and FN1. TRPV3 and ITPR3 were found interacting with EGFR in the network generated from the KMIO brown module dataset. In the network from the GSE32424 turquoise module dataset, 13 ion channels, TRPV3, GJB1, GJC1, P2RX4, ITPR3, TRPC6, RYR2, CLIC4, LRRC8A, TRPC1, GJA5, TRPV2, and KCNMA1, interacted with seven hub nodes that included EGFR, CDH2, IL6, AKT1, CAV1, EP300, and IGF1. VDAC2, ITPR2, VDAC1, GJB2, and CFTR were found interacting with HSPA4, MAPK3, and CDH1 in the network from the GSE32424 blue module (Table 2).

**TABLE 2:** List of ion channels interacting with hub nodes in the protein–protein interaction networks.

PPIN	Hub nodes interacting with ion channels	Hub node category	Ion channels interacting with a hub node
KMIO—blue module PPIN	AKT1	EMT- and lipid metabolism-related genes	GJA1
	CCL2	EMT-related genes	GJA1 and P2RX4
	CDH2	EMT-related genes	GJA1 and GJC1
	FN1	EMT-related genes	GJA1
KMIO—brown module PPIN	EGFR	EMT-related genes	TRPV3 and ITPR3
GSE32424—turquoise module PPIN	EGFR	EMT-related genes	TRPV3, GJB1, and ITPR3
	CDH2	EMT-related genes	GJC1, TRPC6, and GJA5
	IL6	EMT-related genes	P2RX4
	AKT1	EMT- and lipid metabolism-related genes	ITPR3 and KCNMA1
	CAV1	EMT- and lipid metabolism-related genes	ITPR3, RYR2, CLIC4, LRRC8A, TRPC1, GJA5, and KCNMA1
	EP300	EMT- and lipid metabolism-related genes	TRPC6
	IGF1	EMT-related genes	TRPV2
GSE32424—blue module PPIN	HSPA4	EMT-related genes	VDAC1, VDAC2, and ITPR2
	MAPK3	EMT-related genes	VDAC1
	CDH1	EMT-related genes	GJB2 and CFTR

**FIGURE 5:**
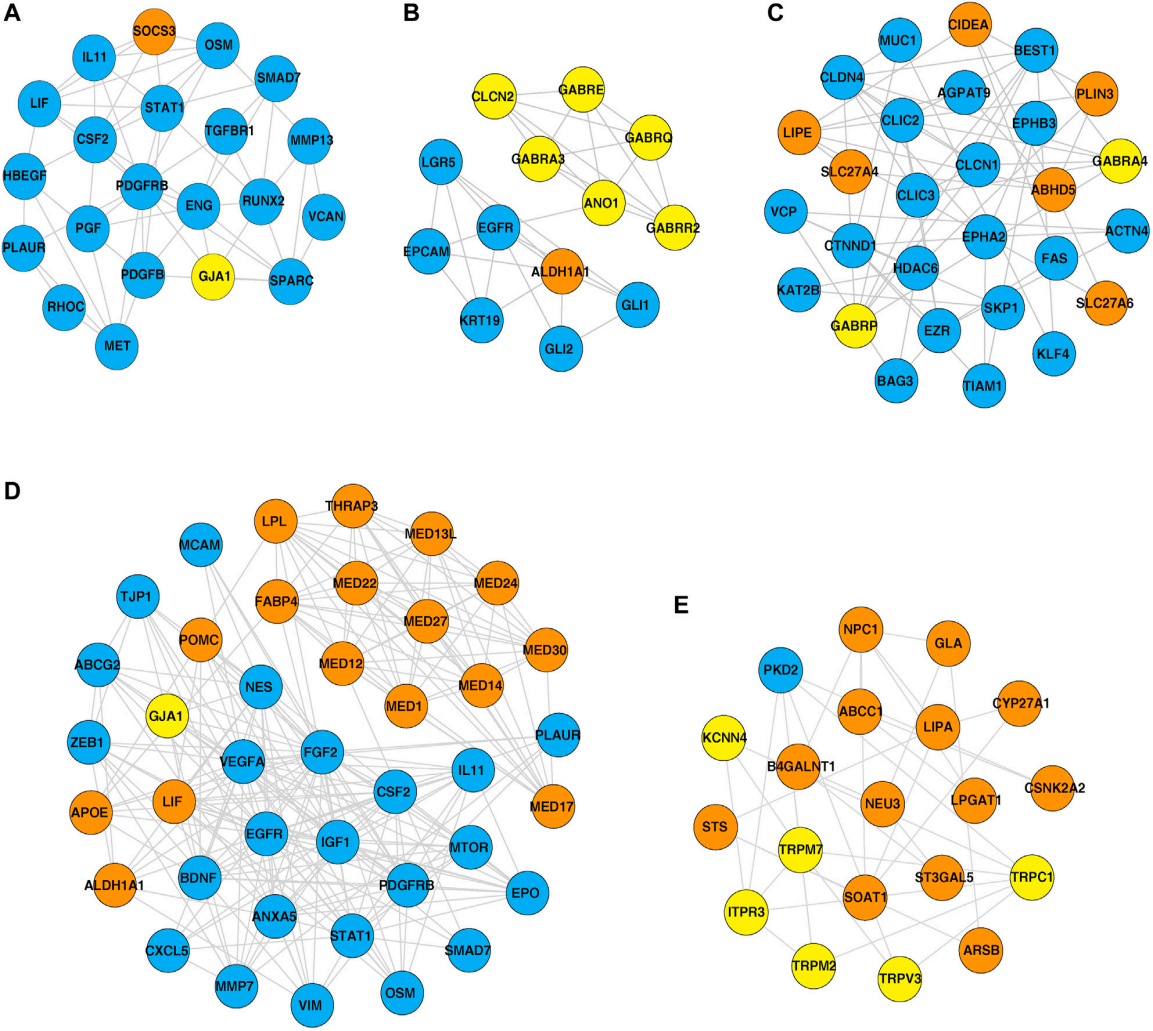
Highly interacting clusters derived from protein–protein interaction networks. Yellow nodes represent ion channels, orange nodes represent lipid metabolism-related proteins, and blue nodes represent EMT-related proteins. **(A,B)** Highly interacting clusters from IOB-KMIO modules. **(C–E)** Highly interacting clusters from the GSE32424 module.

Furthermore, PPI networks were analyzed using the MCODE algorithm to identify dense networks within the PPI networks. In total, 11 clusters in the IOB-KMIO blue module PPI network, 10 clusters in the IOB-KMIO brown module PPI network, 26 clusters in the GSE32424 turquoise module PPI network, and 20 clusters in the GSE32424 blue module PPI network were identified. These network clusters represented the highly interacting protein clusters within the PPINs. Out of these, five clusters—one from the IOB-KMIO blue module, one from the IOB-KMIO brown module, two from the GSE32424 turquoise module, and one from the GSE32424 blue module—comprised ion channels together with lipid metabolism- and EMT-related proteins (Figures 5A–E) (Supplementary Table S11). Several ion channels present in the cluster including *GABRR2*, *ANO1*, and *TRPV3* were found to be negatively correlated with GS76 and positively correlated with both MLR and KS scores, indicating that these ion channels have a higher probability toward stabilizing/acquiring a mesenchymal phenotype. Similarly, *GABRQ*, *GABRA3*, and *TRPM7* negatively correlated with GS76 and positively correlated with MLR and KS scores, indicating that they may have a higher affinity toward maintaining the epithelial phenotype (Supplementary Table S9).

### 3.5 Pathway map of identified altered ion channels, lipid metabolism-related, and EMT-related genes

**FIGURE 6:**
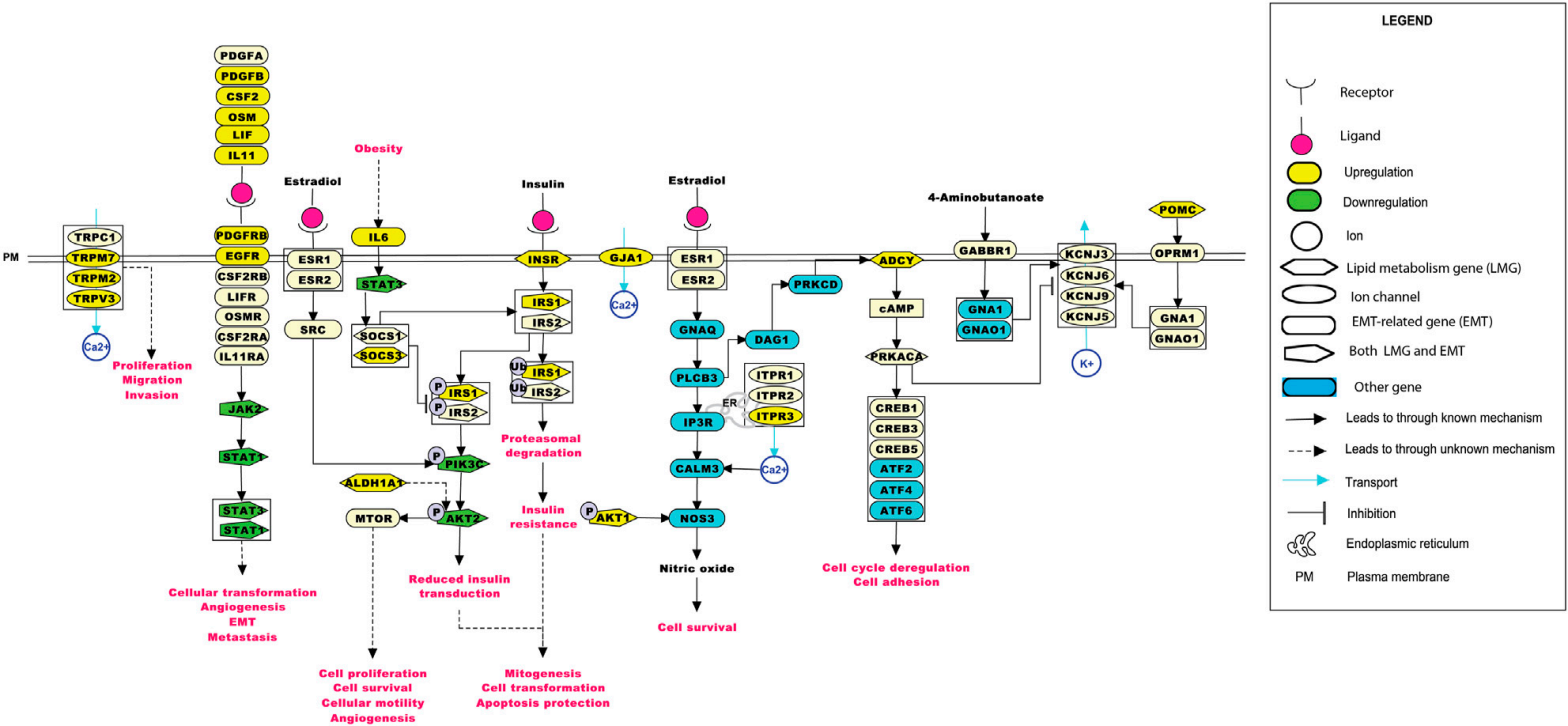
Depiction of events that may occur in ESCC patients upon the deregulation of ion channels with lipid metabolism- and EMT-related genes. The pathway map was generated using PathVisio (v3.3.0). Alterations in any of the molecules could result in deregulations in important cellular processes, such as calcium signaling, insulin resistance, estrogen signaling, and the JAK-STAT pathway.

The significance of the interactions between ion channels, lipid metabolism- and EMT-related genes in various aspects of ESCC tumorigenesis can be laid down by understanding their role in cellular processes. Pathway maps enable the depiction of these cellular processes that may aid in better understanding the disease etiology (Sharma et al., 2020). Figure 6 summarizes the reaction events that are likely to occur in ESCC patients through interactions between deregulated ion channels, lipid metabolism- and EMT-related genes. The pathway map comprises of 77 molecules and 46 reactions. Key cellular processes, including insulin signaling and estrogen receptor-calcium signaling pathways deregulated in ESCC, are depicted in the pathway map.

### 3.6 Prognostically significant ion channels present in the highly interacting clusters

The deregulated ion channels in IOB-KMIO and GSE32424 were compared with the deregulated ion channels in TCGA-ESCC dataset. In total, 240 ion channels were found deregulated in TCGA-ESCC dataset, of which 18 were found deregulated in IOB-KMIO and 49 were present in the deregulated list of ion channels from the GSE32424 dataset. In total, 11 ion channels, *GJB2, GRIN2D, TPCN1, KCTD6, RYR2*, *HTR3B, SCNN1B, CLIC3, KCNE3, CNGA1*, and *CLCN1*, were identified as deregulated in all three datasets (Supplementary Figure S6). Out of 16 ion channels that interacted with EMT- and lipid metabolism-related genes, 14 were found deregulated in at least two datasets. *TRPM2* and *CLCN1* were found deregulated in all three datasets (Supplementary Table S12).

**FIGURE 7:**
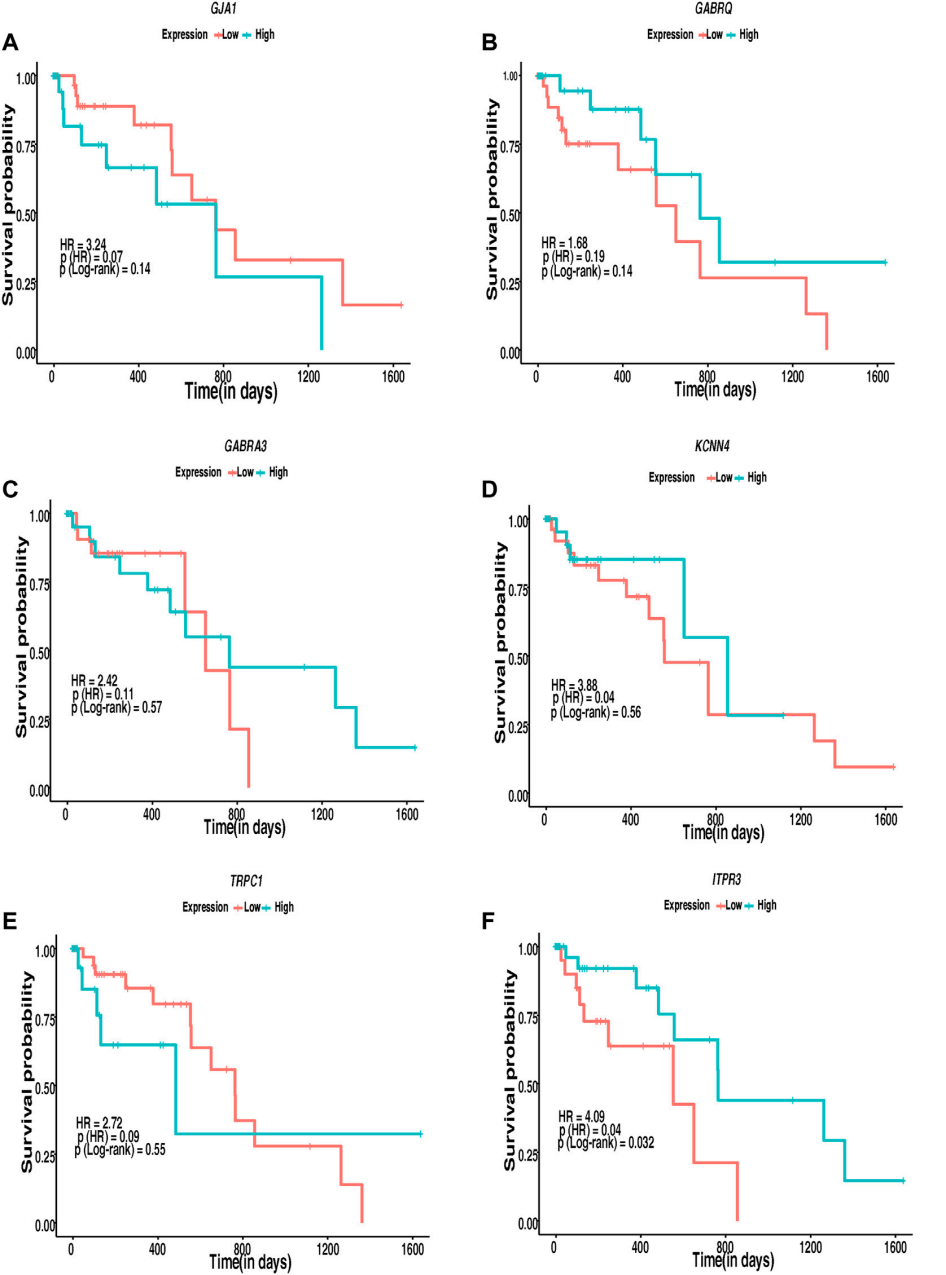
Kaplan–Meier’s 5-year survival curves representing the prognostic relationship between high and low expression of ion channels identified to be interacting with lipid metabolism- and EMT-related genes with survival probability. **(A)** *GJA1*, **(B)** *GABRQ*, **(C)** *GABRA3*, **(D)** *KCNN4*, **(E)** *TRPC1*, and **(F)** *ITPR3.*

The interconnection of ion channels with the survival of patients with ESCC was estimated through KM plots and Cox proportional hazard regression analysis. Ion channels with HR > 1 were selected as major ion channels for the survival of ESCC patients (Figure 7). Six ion channels—*GJA1* [HR: 3.24, *p* (HR): 0.07], *GABRQ* [HR: 1.68, *p* (HR): 0.19], *GABRA3* [HR: 2.42, *p* (HR): 0.11], *KCNN4* [HR: 3.88, *p* (HR): 0.04], *TRPC1* [HR: 2.72, *p* (HR): 0.09], and *ITPR3* [HR: 4.09, *p*(HR): 0.04]—exhibited HR > 1. Patients with high expression of *ITPR3* (*p*-value: 0.03) had a better survival rate. Additionally, 782 gene–drug interactions were identified between the 16 ion channels—*ANO1, CLCN1, CLCN2, GABRA3, GABRA4, GABRE, GABRP, GABRQ, GABRR2, GJA1, ITPR3, KCNN4, TRPC1, TRPM2, TRPM7*, and *TRPV3* drugs (Supplementary Table 13).

## 4 Discussion

Alterations in ion channels affect various cellular processes, such as cell volume regulation, migration, and cell proliferation, that act as a form of sustenance in tumorigenesis (Lang and Stournaras, 2014; Comes et al., 2015). Lipid metabolism is a complex process that involves lipid intake, transport, synthesis, and destruction. Diverse signaling pathways control the biosynthesis and breakdown of different lipids under physiological and pathological conditions (Huang and Freter, 2015). These signaling pathways can either be activated or inhibited depending on the needs of the cell and on responses to the changing cellular environment (Huang and Freter, 2015). Furthermore, the EMT-related genes that form the basis of a metastatic transition in tumor cells are associated with the functionalities of both the ion channels and the lipid mediators. Alterations in these molecules may impact the interactions between proteins, resulting in deregulations of the downstream processes. This could activate a cascade of uncontrolled reactions within the cells in a tissue. Elucidating the role of ion channels with lipid metabolism and EMT would aid future studies to come up with better treatment options for the management of ESCC.

The current study aimed to identify potential altered ion channels interacting with lipid metabolism- and EMT-related genes in ESCC. Differentially expressed ion channels, lipid metabolism- and EMT-related genes from the transcriptomic profiles of patients with ESCC were identified. Furthermore, WGCNA led to the identification of significantly co-expressed gene modules that included the three sets of genes. The correlation of the genes in each module with EMT was obtained using EMT scoring techniques that were consistent across the dataset modules. PPI networks further aided in the identification of PPIs among the differentially co-expressed gene modules. Several ion channels were found to interact with the hub proteins in the network.

Three ion channels GJA1, TRPV3, and ITPR3 were found interacting with the hub proteins in the network and clustered together with lipid metabolism- and EMT-related proteins in the highly interacting clusters.

GJA1 interacted with the hub proteins AKT1, CCL2, CDH2, and FN1 and with the other proteins—SOCS3, POMC, MED13L, MED30, VIM, MED24, MED12, THRAP3, MED1, MED27, MED17, aldehyde dehydrogenase 1 family member A1 (ALDH1A1), APOE, mTOR, LIF, LPL, MED22, FABP4, and MED14 (Figures 4A, 5A, D). GJA1 is the key connexin member present in the immune system and is ubiquitously expressed (Gleisner et al., 2017). It interacts with diverse proteins and can affect cancer cell phenotypes, including cell growth and metastasis (Wang et al., 2018; Aasen et al., 2019). The role of GJA1 has been identified in breast cancer bone metastasis (Wang et al., 2018); its interactions with p21-activated protein kinase 1 to activate mitogen-activated protein kinase (MAPK) signaling in HeLa cells (Aasen et al., 2019) and its upregulation leading to micro-metastasis and tumor vasculature in tumor cell–endothelial cell contact areas (Elzarrad et al., 2008) lead to evidence of GJA1 as a potential target.

TRPV3 interacted with the hub protein EGFR and other proteins—ARSB, NPC1, SOAT1, GLA, LPGAT1, LIPA, CSNK2A2, STS, ST3GAL5, CYP27A1, B4GALNT1, NEU3, and ABCC1 (Figures 4B, 5E). TRP channels, such as TRPV1, TRPV3, and TRPV4, can function as ion transport channels, secondary transducers of ionotropic or metabotropic receptors, or main detectors of physical and chemical stimuli (Holzer, 2011). *TRPV3* was among the TRP channels found upregulated in the study carried out to estimate the immune microenvironment and prognostic effect of TRP channels in patients with ESCC (Zhao et al., 2022). In non-small cell lung carcinoma, the inhibition of TRPV3 led to the reduction in Ca^2+^ ions that arrested the lung cancer cells at the G1/S stage; reduced the expression of cyclinA, cyclinD1, cyclinE, and p-CaMKII; and increased the P27 level (Li et al., 2016). The association between 43 fatty acid metabolism genes and genetic variability in colorectal cancer indicated *TRPV3* with four fatty acid metabolism genes to be associated with a higher risk of colorectal tumor progression (Hoeft et al., 2010), (Holzer, 2011).

ITPR3 was found interacting with EGFR, AKT1, CAV1, ARSB, NPC1, SOAT1, GLA, LIPA, CSNK2A2, LPGAT1, STS, ST3GAL5, CYP27A1, B4GALNT1, NEU3, and ABCC1 (Figures 4B, 5E). ITPR3 dysregulation may interfere with processes leading to endoplasmic reticulum stress (Zheng et al., 2022). The upregulation of *ITPR3* was observed in several malignancies, such as cholangiocarcinoma (Ueasilamongkol et al., 2020), colon cancer (Shibao et al., 2010), melanoma (Kuchay et al., 2017), colorectal cancer (Shibao et al., 2010), cervical squamous cell carcinoma cancer (Yang et al., 2017), glioblastoma (Kang et al., 2010), hepatocellular carcinoma (HCC) (Guerra et al., 2019), mesothelioma, and prostate cancer (Bononi et al., 2017).The upregulation of *ITPR3* plays an essential role in T-lymphocyte apoptosis (Khan et al., 1996). The interactions of ion channels with lipid mediators could possibly be an indication of their role in the dysregulated cascade of events in tumorigenic environments. Pathways contributing to ESCC tumorigenesis through ion channels that interacted with lipid metabolism- and EMT-related proteins included insulin resistance and estrogen receptor-calcium signaling (Figure 6).

### 4.1 Insulin resistance

GJA1 was found interacting with a suppressor of cytokine signaling 3 (SOCS3) and other proteins involved in the mechanism leading to tumor progression through insulin resistance-related pathways. Obesity is one of the major risk factors contributing toward the onset of ESCC (Etemadi et al., 2012). Elevated levels of IL-6 observed in obese individuals could lead to an increase in the levels of suppressor of cytokine signaling 1 (SOCS1) and SOCS3 in white adipose tissue and the liver. At the molecular level, this increase could impair the insulin functionality by binding of the SOCS proteins to insulin receptors, IRS1 and IRS2. Another mechanism leading to the development of insulin resistance by overexpression of the SOCS protein was through reduction in the tyrosine phosphorylation of IRS1 and IRS2 required for insulin transduction (Wunderlich et al., 2013). This signal transduction is carried out through phosphoinositide 3-kinases (PI3K) and the MAPK pathway accounting for mitogenesis, transformation, and apoptosis protection in tumor cells (Arcidiacono et al., 2021). Wunderlich and others have shown the involvement of SOCS1 and SOCS3 in insulin resistance through the elevation of interleukin-6 (IL-6), thus leading to the activation of the JAK-STAT pathway.

### 4.2 JAK/STAT pathway

GJA1 was found interacting with the signal transducer and activator of transcription (STAT), and other proteins involved in this pathway. STAT3 aberrant activation could lead to esophageal cellular transformation, angiogenesis, EMT, and metastasis. Sugase and colleagues have identified the association of the JAK/STAT pathway in the prognosis of patients with ESCC through immunohistochemistry studies (Sugase et al., 2017). The correlation between the phosphorylation levels of STAT3 and JAK1 was significant in predicting the poor prognosis of ESCC patients, indicating their importance in better management of ESCC (Rah et al., 2022).

### 4.3 PI3K-AKT pathway

ALDH1A1 was found to interact with the ion channels GABRE, GABRR2, GABRQ, GABRA3, ANO1, and GJA1 (Figure 5B). The PI3K-AKT pathway known to be deregulated in ESCC is activated by the overexpression of ALDH1A1. ALDH1A1 increases the expression of AKT serine/threonine kinase 1 (AKT1) and *p*-AKT. The phosphorylation of AKT activates several substrates that regulate cellular proliferation, survival, and motility (Shi et al., 2019). ALDH1A1 plays a significant role in maintaining stem cell properties in patients with ESCC through the AKT pathway. ALDH1A1 could be a potential target in patients with ESCC (Wang et al., 2020).

### 4.4 Estrogen receptor-Ca^2+^ signaling pathway

The estrogen receptor-Ca^2+^ signaling pathway was found to be associated with male predominance of ESCC. The estrogen receptors present on the plasma membrane activate multiple intracellular secondary messengers, including Ca^2+^ signaling. An increase in the cytoplasmic Ca^2+^ levels occur due to the release of Ca^2+^ from the endoplasmic reticulum through ion channels, such as ITPR3 (Zhang et al., 2017).

Cox proportional regression analysis with high HR indicated the alterations in the ion channels *GJA1*, *GABRQ*, *GABRA3*, *KCNN4*, *TRPC1*, and *ITPR3* to possibly be associated with poor survival in patients with ESCC. Patients with low expression of *GJA1* (*p*-value: 0.14), *KCNN4* (*p*-value: 0.56), and *TRPC1* (*p*-value: 0.55) and higher expression of *GABRQ* (*p*-value: 0.14), *GABRA3* (*p*-value: 0.57), and *ITPR3* (*p*-value: 0.032) may show better survival. However, the number of patients with ESCC was limited to attain statistical significance to support the expression difference using a log rank *p*-value <0.05 in survival analysis except *ITPR3*. Of these, GJA1 and ITPR3 were found interacting with hub proteins in the interactome and with other proteins in the highly interacting network clusters. Several ion channels found deregulated in the present study, including *GJA1*, and *ITPR3*, interacted with various known drugs. The presence of ion channels on the plasma membrane makes them accessible targets to therapeutic drugs. Moreover, their putative significance as diagnostic and prognostic markers and the availability of multiple drugs interacting with the potential ion channels make them ideal targets for clinical studies. *In vivo* mouse model studies showed that SKF96365, a phenylethylimidazole, and an inhibitor of store operated calcium channels (SOCE) resulted in decreased tumor volume by 50% in 4 weeks (Leanza et al., 2016). The inhibition of *TRPC6* in ESCC cells leads to the suppression of proliferation and induction of the G2/M phase arrest (Zhang et al., 2018). The inhibition of *TRPV2* by tranilast; inhibition of *ANO1* by T16Ainh-A01, digallic acid, and tannic acids; and the inhibition of voltage-gated sodium channels by valproic acid were reported as potential targeted therapeutic agents in esophageal squamous cell carcinoma (Shiozaki et al., 2018; Capatina et al., 2022; Bill et al., 2014; Wheler et al., 2014). However, these channels are ubiquitously present, and toxicity toward normal cells on targeting these channels has not been addressed in depth. Further *in vivo* and *in vitro* studies could be carried out to elucidate the role of identified potentially altered ion channels interacting with genes of lipid metabolism- and EMT-related genes.

## 5 Conclusion

The interactome of ion channels with lipid metabolism- and EMT-related genes of patients with ESCC determined potential altered ion channels in ESCC. Furthermore, altered ion channels were found to be involved in several pathways, including insulin resistance, JAK-STAT, PI3K-AKT, and estrogen receptor-Ca^2+^ signaling. Additionally, *ITPR3* was associated with poor prognosis of patients with ESCC. Targeting potential ion channel networks with inhibitors could most likely be an alternative therapeutic strategy to improve ESCC patient care. Moreover, experimental studies demonstrating the mechanistic view of these interactions need to be established.

## Data Availability

The datasets presented in this study can be found in online repositories. The names of the repository/repositories and accession number(s) can be found at: https://www.ncbi.nlm.nih.gov/, PRJNA955681. Customized R scripts used for identification of DEGs, co-expressed gene modules and survival analysis and the ESCC ICs-LMG-EMT pathway reaction data are available in the GPML format through the GitHub repository via the following URLs: https://github.com/js-iob/ESCC_ICs-LMG-EMT/blob/main/differential_expression_ESCC.R, https://github.com/js-iob/ESCC_ICs-LMG-EMT/blob/main/WGCNA_ESCC.R, https://github.com/js-iob/ESCC_ICs-LMG-EMT/blob/main/survival_ESCC.R, https://github.com/js-iob/ESCC_ICs-LMG-EMT/blob/main/ESCC_ICs-LMG-EMT_PathwayMap.gpml.
