# Supplementary material for: Aberrations in ion channels interacting with lipid metabolism and epithelial–mesenchymal transition in esophageal squamous cell carcinoma
Source: Front Mol Biosci. 2023 Jul 17;10:1201459. doi: 10.3389/fmolb.2023.1201459 (PMC10388552; doi:10.3389/fmolb.2023.1201459)
Supplement: Supplementary file 4 [file DataSheet2.docx]

Supplementary Material

**Aberrations in ion channels interacting with lipid metabolism and epithelial mesenchymal transition in esophageal squamous cell carcinoma**

K. T. Shreya Parthasarathi, Susmita Mandal, John Philip George, Kiran Bharat Gaikwad, Sruthi Sasidharan, Seetaramanjaneyulu Gundimeda, Mohit Kumar Jolly, Akhilesh Pandey and Jyoti Sharma*

***Correspondence:**

Dr. Jyoti Sharma: jyoti@ibioinformatics.org

# Supplementary Tables and Figures

## Supplementary Tables

**Supplementary table 2: Clinical Information of ESCC patients from Kidwai Memorial Institute of Oncology (KMIO)**

|  | **Total number of samples** |
| --- | --- |
| **Number of Individuals** | 12 |
| **Average Age (Years)** | 53 |
| **Gender** |  |
| Female | 6 |
| Male | 6 |
| **Sample type** |  |
| Primary tumor | 12 |
| Adjacent normal | 12 |
| **Tumor grade** |  |
| Unknown | 1 |
| Grade 2 | 8 |
| Grade 3 | 3 |

**Supplementary table 1: Total number of genes included in the study**

| **Gene type** | **Total** |
| --- | --- |
| Ion channels | 374 |
| EMT-related genes | 1184 |
| Lipid metabolism genes | 1087 |

**Supplementary table 3: Clinical Information of ESCC patients from GSE32424 dataset**

|  | **Total number of samples** |
| --- | --- |
| **Number of Individuals** | 9 |
| **Average Age (Years)** | 58 |
| **Gender** |  |
| Female | 1 |
| Male | 8 |
| **Sample type** |  |
| Primary tumor | 7 |
| Non tumor | 5 |
| **Lymph node metastasis** |  |
| Yes | 4 |
| No | 5 |
| **TNM Stage** |  |
| T2N0M0 | 5 |
| T2N1M0 | 1 |
| T3N1M0 | 3 |

**Supplementary table 4: Clinical Information of ESCC patients from TCGA datasets**

|  | **Total number of samples** |
| --- | --- |
| **Number of Individuals** | 94 |
| **Average Age (Years)** | 58 |
| **Gender** |  |
| Female | 14 |
| Male | 80 |
| **Sample type** |  |
| Primary tumor | 82 |
| Solid tissue normal | 12 |
| **Vital status** |  |
| Alive | 56 |
| Dead | 24 |
| **Pathological stage** |  |
| Stage IA | 3 |
| Stage IB | 4 |
| Stage IIA | 36 |
| Stage IIB | 12 |
| Stage III | 10 |
| Stage IIIA | 8 |
| Stage IIIB | 4 |
| Stage IV | 2 |
| Stage IVA | 1 |
| Unknown | 2 |

**Supplementary table 5: Criteria used for the selection of normal samples from GTEx**

| **Filtering criteria** | **Total samples** | **Male** | **Female** |
| --- | --- | --- | --- |
| No filter | 653 | 416 | 237 |
| Filtered out gastroesophageal junction samples | 516 | 327 | 189 |
| Filtered out samples belonging to the age group under 30 | 459 | 292 | 167 |
| Filtered out samples whose cause of death was due to serious ailment or cancer | 100 | 74 | 26 |
| Filtered out randomly based on male:female ratio to equalise with TCGA-ESCC samples | 70 | 60 | 10 |

**Supplementary table 6: Number of reads from KMIO mapped to reference genome using STAR**

| **Sample id** | **Uniquely mapped reads (in %)** | **% of reads mapped to multiple loci** | **Unmapped reads: too short** |
| --- | --- | --- | --- |
| 1493_13 – N | 80.44% | 12.51% | 6.57% |
| 1493_13 – T | 85.91% | 8.68% | 4.90% |
| 3507_15 – N | 84.67% | 8.99% | 5.85% |
| 3507_15 – T | 85.03% | 9.92% | 4.58% |
| 3914_12 – N | 78.41% | 14.87% | 6.17% |
| 3914_12 – T | 68.73% | 22.94% | 7.86% |
| 4111_15 – N | 76.39% | 16.84% | 6.31% |
| 4111_15 – T | 85.60% | 8.70% | 5.19% |
| 7708_15 – N | 87.12% | 6.64% | 5.81% |
| 7708_15 – T | 73.06% | 17.94% | 8.75% |
| 14228_13 – N | 76.02% | 17.12% | 6.40% |
| 14228_13 – T | 74.97% | 18.12% | 6.42% |
| 4074_15 – N | 88.63% | 5.42% | 5.66% |
| 4074_15 – T | 86.47% | 6.45% | 6.73% |
| 5212_15 – N | 84.89% | 6.37% | 8.45% |
| 5212_15 – T | 87.07% | 5.71% | 6.87% |
| 6067_11 – N | 78.32% | 8.35% | 13.07% |
| 6067_11 – T | 83.17% | 8.33% | 8.18% |
| 7825_15 – N | 86.35% | 6.42% | 6.88% |
| 7825_15 – T | 82.18% | 8.43% | 9.06% |
| 11858_17 – N | 87.23% | 5.22% | 7.11% |
| 11858_17 – T | 85.13% | 7.73% | 6.81% |
| 14423_14 – N | 89.05% | 5.75% | 4.91% |
| 14423_14 – T | 87.21% | 7.06% | 5.44% |

**Supplementary table 7: Number of reads from GSE32424 mapped to reference genome using STAR**

| **Sample id** | **Uniquely mapped reads (in %)** | **% of reads mapped to multiple loci** | **Unmapped reads: too short** |
| --- | --- | --- | --- |
| GSM802457_1T | 51.61% | 39.46% | 0.11% |
| GSM802458_2T | 65.32% | 29.96% | 0.13% |
| GSM802459_3T | 64.14% | 29.69% | 0.16% |
| GSM802460_6T | 52.55% | 26.18% | 0.12% |
| GSM802461_7T | 56.41% | 26.27% | 0.18% |
| GSM802462_8T | 57.73% | 25.13% | 0.13% |
| GSM802463_9T | 50.39% | 30.52% | 0.15% |
| GSM802464_4N | 58.42% | 34.39% | 0.21% |
| GSM802465_5N | 62.40% | 27.42% | 0.18% |
| GSM802466_6N | 49.04% | 34.73% | 0.15% |
| GSM802467_8N | 59.94% | 26.86% | 0.23% |
| GSM802468_9N | 56.49% | 29.53% | 0.21% |

**Supplementary table 8: List of samples present in IOB-KMIO dataset, their corresponding EMT score estimated using GS76, MLR and KS EMT scoring methods and correlation of expression values of genes obtained in the WGCNA selected modules with GS76, MLR and KS EMT scoring methods (Excel sheet)**

**Supplementary table 9: Correlation between GS76, MLR and KS EMT scoring methods across samples**

| **Dataset** | **76GS.MLR_Cor** | **76GS.MLR_Pval** | **76GS.KS_Cor** | **76GS.KS_Pval** | **KS.MLR_Cor** | **KS.MLR_Pval** |
| --- | --- | --- | --- | --- | --- | --- |
| IOB-KMIO | 0.8372123 | 3.38E-07 | -0.6412812 | 0.0007328 | 0.8145619 | 1.27E-06 |

**Supplementary table 10: List of proteins found in clusters generated by MCODE**

| MCODE cluster | Ion channels | EMT-related proteins | Lipid metabolism proteins |
| --- | --- | --- | --- |
| IOB-KMIO_blue | GJA1 | MET, SMAD7, LIF, SPARC, VCAN, CSF2, PDGFRB, STAT1, RHOC, TGFBR1, PLAUR, MMP13, PGF, ENG, PDGFB, IL11, HBEGF, RUNX2, OSM | SOCS3 |
| IOB-KMIO_brown | GABRE, GABRR2, GABRQ, GABRA3, ANO1 | GLI1, EGFR, GLI2, LGR5, CLCN2, EPCAM, KRT19 | ALDH1A1 |
| GSE32424_turquoise1 | GJA1 | ANXA5, TJP1, CXCL5, PDGFRB, CSF2, FGF2, VIM, ZEB1, ABCG2, EPO, BDNF, IGF1, OSM, EGFR, SMAD7, MTOR, MCAM, VEGFA, STAT1, PLAUR, MMP7, NES, IL11 | POMC, MED13L, MED30, VIM, MED24, MED12, THRAP3, MED1, MED27, MED17, ALDH1A1, APOE, MTOR, LIF, LPL, MED22, FABP4, MED14 |
| GSE32424_turquoise2 | TRPV3, KCNN4, TRPM7, TRPC1, TRPM2, ITPR3 | PKD2 | ARSB, NPC1, SOAT1, GLA, LIPA, CSNK2A2, LPGAT1, STS, ST3GAL5, CYP27A1, B4GALNT1, NEU3, ABCC1 |
| GSE32424_blue | GABRP, GABRA4 | SKP1, MUC1, CLIC3, CTNND1, CLDN4, EPHA2, EPHB3, AGPAT9, KAT2B, KLF4, VCP, CLIC2, BAG3, TIAM1, FAS, HDAC6, EZR, BEST1, CLCN1, ACTN4 | CIDEA, SLC27A4, SLC27A6, LIPE, PLIN3, ABHD5 |

**Supplementary table 11: List of deregulated ion channels interacting with lipid metabolism and EMT related genes in TCGA – ESCC, IOB – KMIO and GSE32424 datasets**

| Genes | TCGA - ESCC | IOB - KMIO | GSE32424 |
| --- | --- | --- | --- |
| *GJA1* | Upregulated | - | Upregulated |
| *GABRE* | Upregulated | - | Upregulated |
| *GABRR2* | - | Upregulated | - |
| *GABRQ* | Upregulated | - | Upregulated |
| *GABRA3* | Upregulated | - | Upregulated |
| *ANO1* | Upregulated | - | Upregulated |
| *CLCN2* | Upregulated | - | Upregulated |
| *TRPV3* | Upregulated | Upregulated | - |
| *KCNN4* | Upregulated | - | Upregulated |
| *TRPM7* | Upregulated | - | Upregulated |
| *TRPC1* | Downregulated | - | Upregulated |
| *TRPM2* | Upregulated | Upregulated | Upregulated |
| *ITPR3* | Upregulated | - | Upregulated |
| *GABRP* | - | - | Downregulated |
| *GABRA4* | Downregulated | Downregulated | - |
| *CLCN1* | Upregulated | Downregulated | Downregulated |

**Supplementary table 12: List of drugs interacting with potential ion channels (Excel sheet)**

## Supplementary Figures


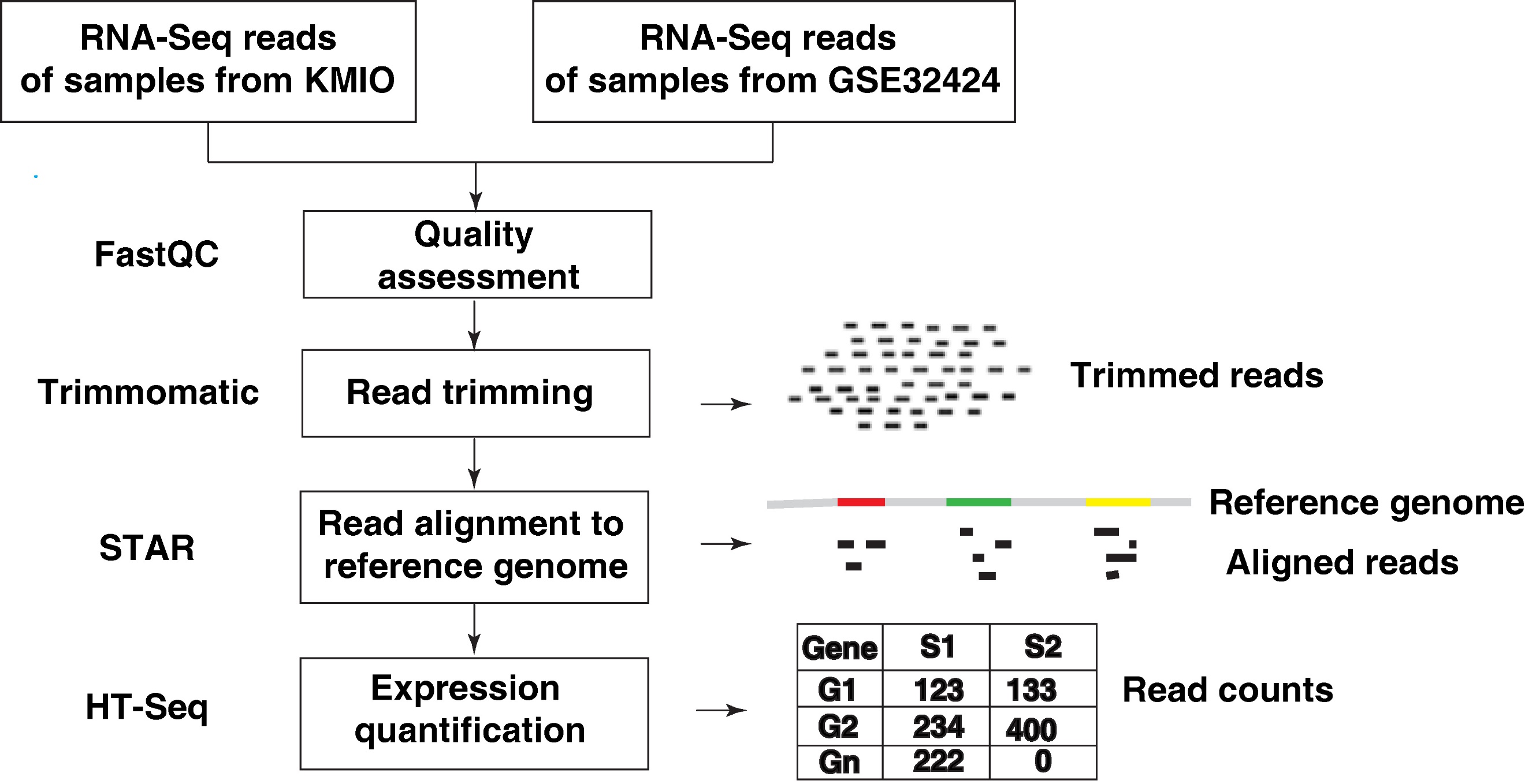


**Supplementary figure 1: RNA-Seq data analysis pipeline**


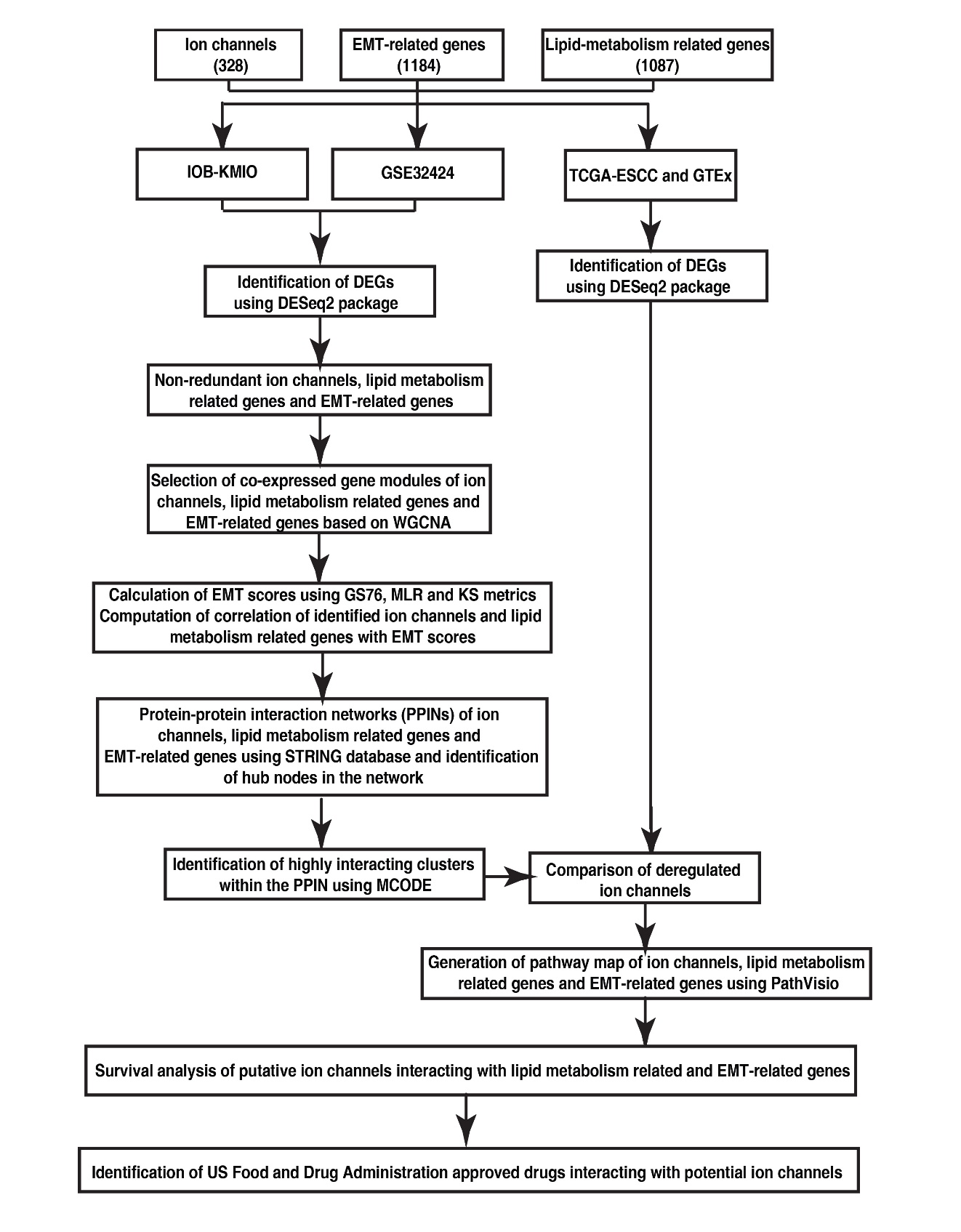


**Supplementary figure 2: Depiction of workflow for the identification of ion channels and their correlation with lipid metabolism and EMT**


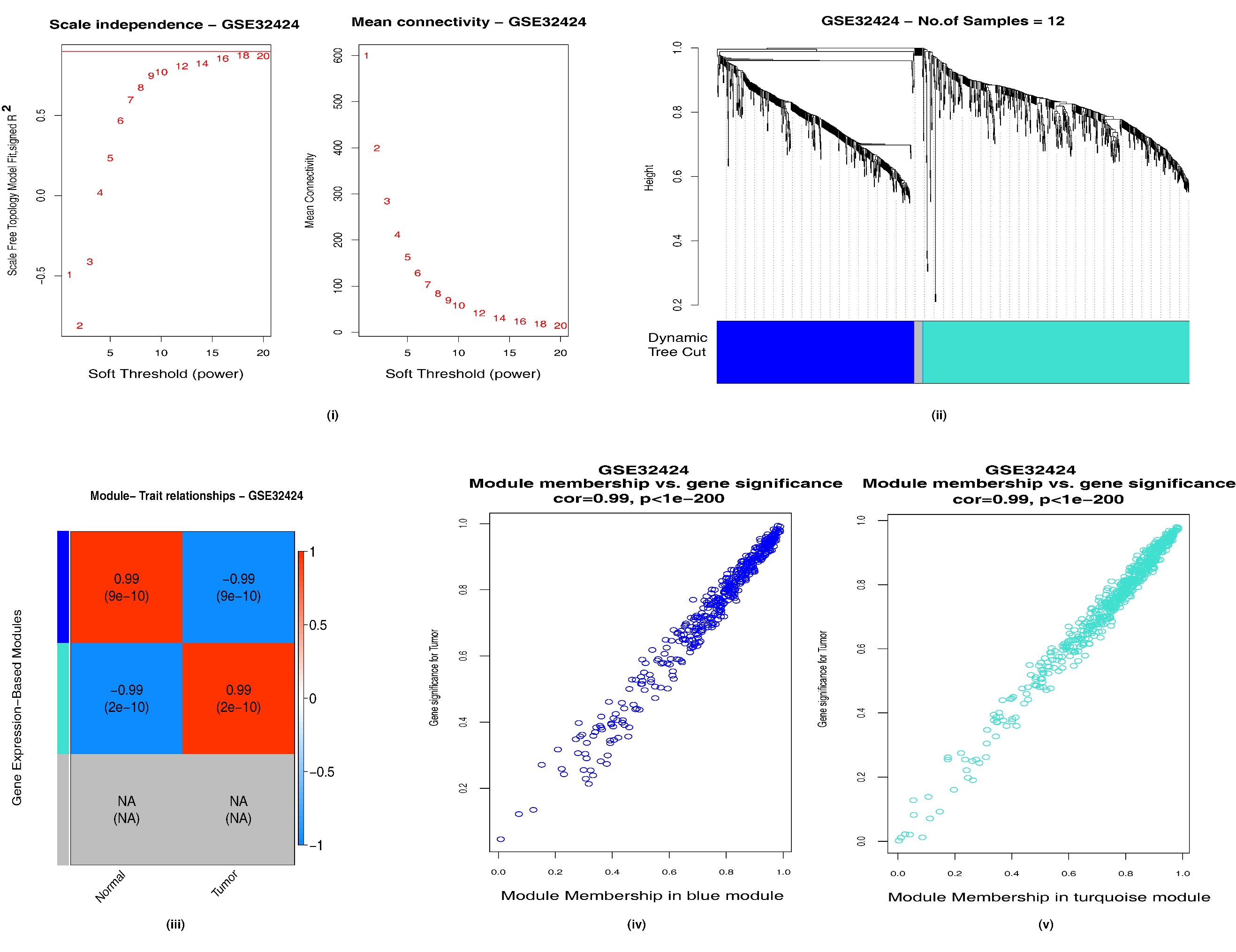


**Supplementary figure 3: Weighted gene co-expression network analysis of GSE32424 dataset. Gene modules with co-expressed ion channels, lipid metabolism genes and EMT-related genes modules based on the non-redundant DEGs. (i) Soft-thresholding power: (i) A soft-thresholding power of 18 was chosen in the dataset GSE32424 to ensure scale-free network model (ii) Hierarchical clustering of genes into modules. The horizontal bar below the tree diagram represent the modules depicted by different colors. 2 modules (turquoise and blue) were obtained (iii) Correlation between module eigengenes and binary traits—normal and tumor. Rows correspond to modules depicted as different colors and columns are the binary traits. Numbers in each cell are the correlation coefficient between module eigengenes and the binary traits and the corresponding p-value. (i) The blue and turquoise modules were chosen as significant modules. (iv) Scatter plot of gene significance (GS) for the binary trait vs. the module membership (MM) in the selected modules. Both blue and turquoise modules showed a better within module gene correlation**


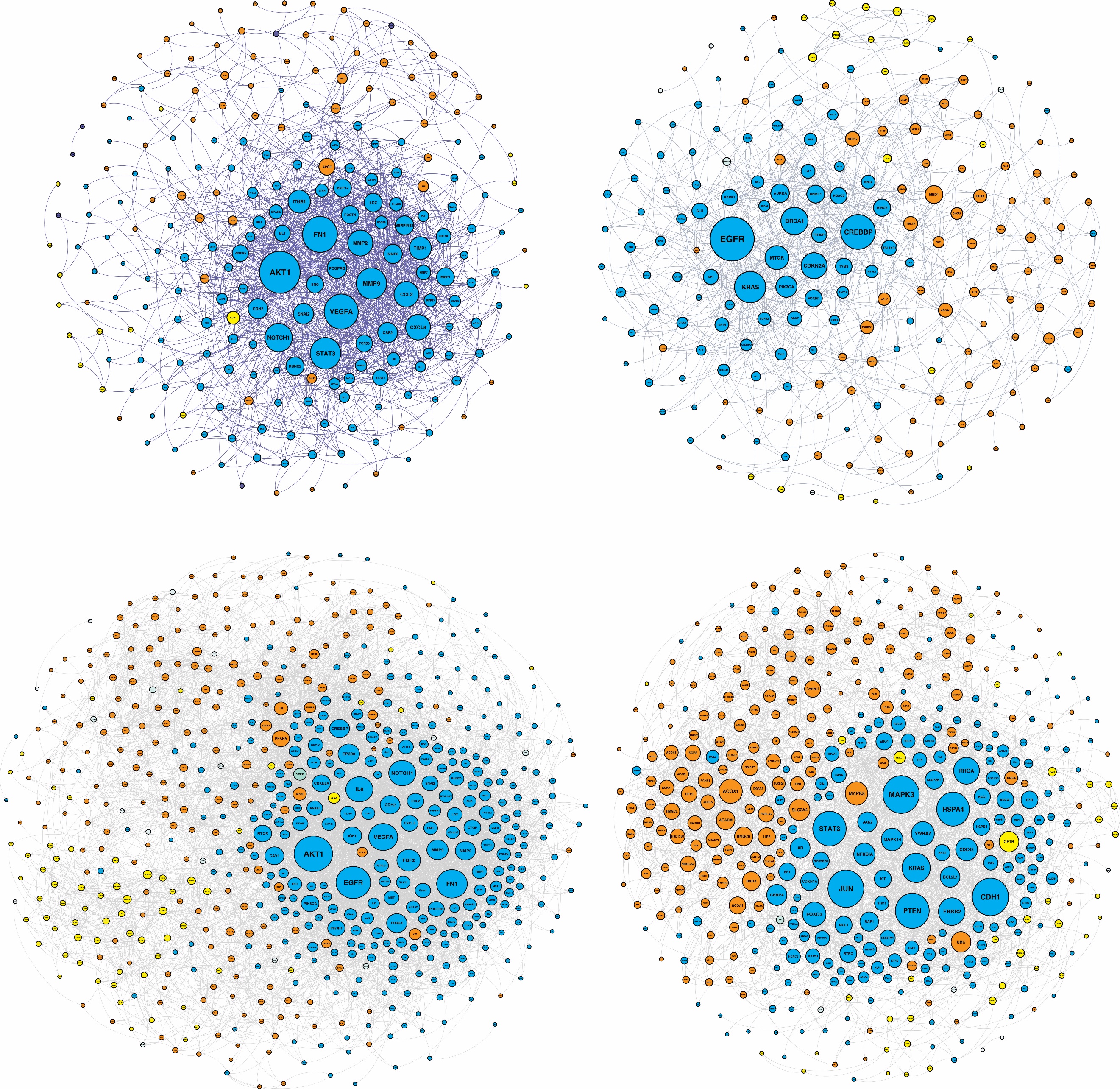


**Supplementary figure 4: Protein-protein interaction networks of significantly co-expressed gene modules.** **Yellow nodes represent ion channels, orange nodes represent lipid metabolism proteins and blue nodes represent EMT-related proteins (i) PPIN of significantly co-expressed genes in blue module of IOB-KMIO dataset (ii) PPIN of significantly co-expressed genes in brown module of IOB-KMIO dataset (iii) PPIN of significantly co-expressed genes in turquoise module of GSE32424 dataset(iv) PPIN of significantly co-expressed genes in blue module of GSE32424 dataset**


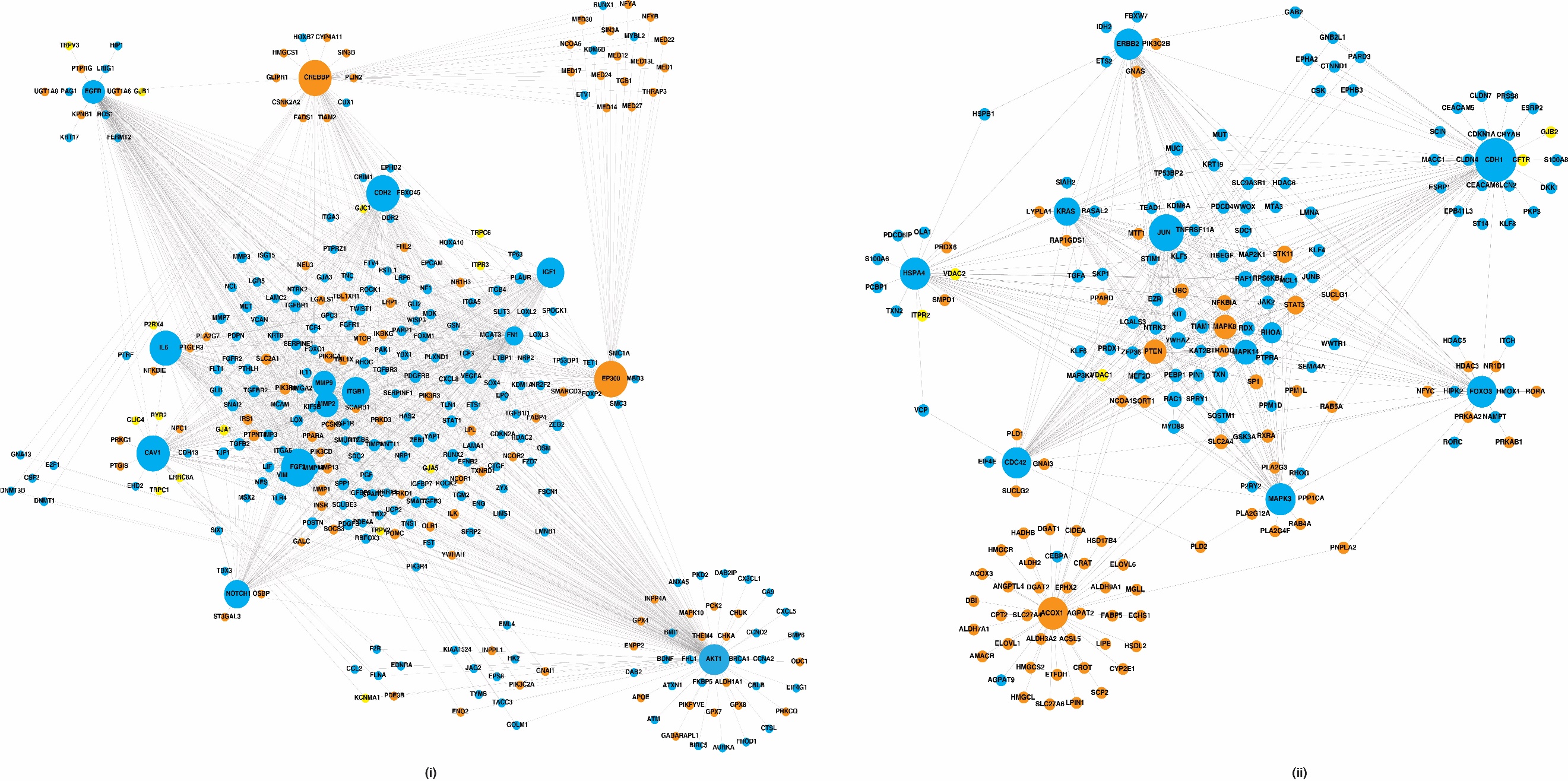


**Supplementary figure 5: Protein – protein interaction network of the hub nodes in the network: Yellow nodes represent ion channels; orange nodes represent lipid metabolism proteins and blue nodes represent EMT-related proteins. (i) PPIN depicting the proteins interacting with the hub nodes in IOB-GSE32424 turquoise module network. (ii) PPIN depicting the proteins interacting with the hub nodes in IOB-GSE32424 blue module network**.


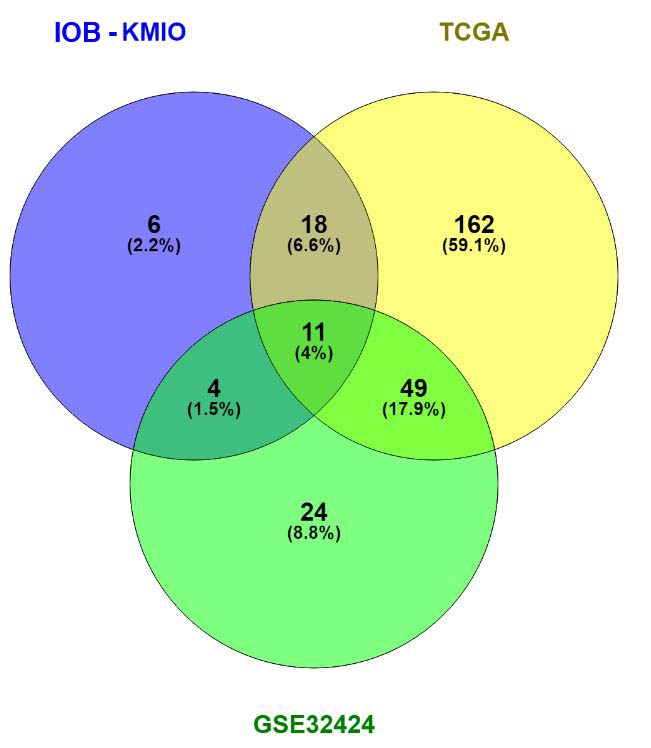


**Supplementary figure 6: Venn diagram depicting the overlaps in the differentially expressed ion channels, TCGA, IOB-KMIO and GSE32424 datasets**
