# Supplementary material for: Aberrations in ion channels interacting with lipid metabolism and epithelial–mesenchymal transition in esophageal squamous cell carcinoma
Source: Front Mol Biosci. 2023 Jul 17;10:1201459. doi: 10.3389/fmolb.2023.1201459 (PMC10388552; doi:10.3389/fmolb.2023.1201459)
Supplement: Supplementary file 5 [file DataSheet1.docx]

Supplementary File 1

**Aberrations in ion channels interacting with lipid metabolism and epithelial mesenchymal transition in esophageal squamous cell carcinoma**

K. T. Shreya Parthasarathi, Susmita Mandal, John Philip George, Kiran Bharat Gaikwad, Sruthi Sasidharan, Seetaramanjaneyulu Gundimeda, Mohit Kumar Jolly, Akhilesh Pandey and Jyoti Sharma*

***Correspondence:**

Dr. Jyoti Sharma: jyoti@ibioinformatics.org

The laboratory procedures of the study samples were performed by MedGenome Labs Ltd, Bangalore.

**RNA Sample extraction and QC**

RNA was extracted from 24 tissue samples by using RNeasy Mini Kit (QIAGEN, Cat# 74104). The 24 Extracted RNA and 12 Direct RNA samples were quantified using Qubit RNA Assay HS (Invitrogen, Cat# Q32852). RNA purity was checked using QIAxpert and RNA integrity was assessed on Tape Station using RNA HS Screen Tapes (Agilent, Cat#
5067-5579). 24 QC passed, shortlisted RNA samples were proceeded for the Total RNA library prep.

**RNA Library Prep Protocol**

NEB Ultra RNA-Seq Library Prep kit protocol was used to prepare libraries for Total RNA sequencing (NEB, Cat#E7530L). First, the ribosomal RNA (rRNA) which constitutes for ~95% of the total RNA population were removed using biotinylated, target-specific oligos combined with Ribo-Cop rRNA removal beads. Following purification, the ribo-depleted RNA was fragmented using divalent cations under elevated temperatures. The cleaved RNA
fragments were copied into first strand cDNA using reverse transcriptase. Second strand cDNA synthesis was performed, using DNA Polymerase I and RNase H enzyme. The cDNA fragments were then subjected to a series of enzymatic steps which repair the ends, tails the 3’ end with a single ‘A’ base, followed by ligation of the adapters. The adapter ligated products were then purified and enriched using the following thermal conditions: initial denaturation 98°C for 30sec; 13 cycles of - 98°C for 10sec, 65°C for 75sec; final extension of 65°C for 5mins. PCR products are then purified and checked for fragment size distribution on
TapeStation using D1000 DNA ScreenTapes (Agilent, Cat# 5067-5582).

**Sequencing protocol:**

Prepared libraries were quantified using Qubit High Sensitivity Assay (Invitrogen, Cat#Q32852). The obtained libraries were pooled and diluted to final optimal loading concentration before cluster amplification on Illumina flow cell. Once the cluster generation was completed, the cluster flow cell was loaded on Illumina HiSeq 4000 instrument to generate 60M, 100bp paired end reads.
